# Clinical and microbial insights into polymicrobial vascular graft infections: a prospective observational study

**DOI:** 10.1007/s15010-026-02775-1

**Published:** 2026-03-26

**Authors:** Catrina I. Pedrett, Jana Epprecht, Bruno Ledergerber, Julian Bär, Judith Bergada-Pijuan, Claudio T. Acevedo, Andrea Tarnutzer, Benedikt Reutersberg, Pascal M. Frey, Annelies S. Zinkernagel, Silvio D. Brugger, Barbara Hasse

**Affiliations:** 1 Department of Infectious Diseases and Hospital Epidemiology, University Hospital Zurich, University of Zurich, Zurich, Switzerland; 2 Department of Vascular Surgery, University Hospital Zurich, University of Zurich, Zurich, Switzerland; 3 Department of General Internal Medicine, Bern University Hospital (Inselspital), Bern, Switzerland

**Keywords:** Vascular graft/endograft infections, Quantitative fitness analysis (QFA), Polymicrobial infections, Microbe-microbe interactions

## Abstract

**Objectives:**

Vascular graft/endograft infections (VGEI) are difficult-to-treat and associated with high morbidity and mortality. Up to 40% of VGEI are polymicrobial, complicating clinical management. This study investigates microbial interactions and their impact on clinical outcomes.

**Methods:**

We analyzed patient characteristics and survival in poly- versus monomicrobial infections from the VASGRA cohort, a Swiss prospective observational study. Kaplan–Meier analysis and Cox proportional hazards models were used to assess survival. Furthermore, a subset of patient-derived bacterial strains underwent Quantitative Fitness Analysis (QFA) to quantify changes in fitness in mono- and polymicrobial conditions.

**Results:**

Among 227 VGEI episodes, 81 (35.7%) were polymicrobial. Overall survival was 87.7% at one year and 77.5% at five years, with polymicrobial infections associated with reduced survival (log-rank *p* = 0.004). Multivariable analyses confirmed that polymicrobial infections (adjusted HR = 2.94, 95% CI: 1.60–5.41, *p* < 0.001) and age per 10 years older (adjusted HR = 2.07, 95% CI: 1.26–3.41, *p* = 0.004) were independent risk factors for increased mortality. There was also a trend of an association with diabetes mellitus (adjusted HR = 1.95, 95% CI: 0.91–4.29, *p* = 0.088). QFA of five strain pairs revealed diverse interaction patterns among pathogens, ranging from complete inhibition to enhanced growth in co-culture.

**Conclusion:**

Polymicrobial infections, along with older age and diabetes mellitus, were identified as independent risk factors for increased mortality in VGEI patients. QFA revealed diverse bacterial interactions, highlighting the complexity of polymicrobial environments in VGEI.

**Supplementary Information:**

The online version contains supplementary material available at https://doi.org/10.1007/s15010-026-02775-1.

## Introduction

Vascular graft/endograft infections (VGEI) are severe complications occurring in 1–6% of vascular procedures [1]. Peripheral graft infections can lead to limb loss and rarely death, while aortic graft infections, though less frequent, have a mortality rate exceeding 20% [1]. Treatment typically involves surgery and antimicrobial therapy [1, 2]. Most VGEIs are caused by coagulase-negative staphylococci, *Staphylococcus aureus*, and enterococci [1–5]. In intracavitary abdominal infections, Enterobacterales are common [5–7], whereas VGEI with secondary aorto-enteric fistula are often polymicrobial and frequently involve fungi [8, 9].

Polymicrobial VGEI occur in 24–41% of cases [3–5, 7, 10]. Polymicrobial infections worsen clinical outcome in prosthetic joint infection, infective endocarditis, and bloodstream infection [11–13]. The impact of polymicrobial communities on the clinical course of VGEI, however, remains unclear. Microorganisms within such communities can alter each other’s gene expression and metabolism, potentially influencing infection progression [14–16]. Quantitative fitness analysis (QFA) is a low-cost, high-throughput method for assessing a strain’s reproductive fitness, either alone or in competition with another strain [17]. This approach is ideal to study microbial interactions in polymicrobial infections.

This project had two aims: to compare clinical characteristics and outcomes in polymicrobial versus monomicrobial VGEI in a clinical cohort study, and to quantify interspecies interactions between different microorganisms isolated from polymicrobial VGEI.

## Materials and methods

### Study design and setting

This study was performed as part of VASGRA, a prospective cohort study at the University Hospital Zurich, Switzerland, enrolling patients aged ≥ 18 years undergoing treatment for VGEI. Physicians and study nurses collected standardized demographic, clinical, surgical, and laboratory data every six months during study visits, with follow-up lasting up to 10 years. Tissue samples from surgical interventions were collected for further use.

For this analysis, participants diagnosed with VGEI from January 2013 to March 2023 with at least one baseline and one follow-up visit were included. VGEI diagnosis followed the Management of Aortic Graft Infection Collaboration criteria [18] requiring either two major criteria or one major and one minor criterion from different categories. Multiple VGEI episodes per patient were recorded separately.

The study was approved by the Institutional Review Board of Zurich, Switzerland (cohort study: KEK-ZH 2012-0583, PB_2016-01320; patient sample processing: KEK-ZH 2017-02225). Informed consent was obtained from all participants.

### Definitions

A VGEI episode was categorized as polymicrobial if ≥ 2 microorganisms were cultured in ≥ 2 samples of perigraft tissue, graft material, or blood cultures. Potential contaminants, such as coagulase-negative staphylococci or *Cutibacterium acnes*, were considered significant if detected in ≥ 3 tissue samples or in both a tissue and a vascular graft sample.

VGEI located in the abdomen or thorax were categorized as intracavitary abdominal or thoracic infections, respectively [1]. A VGEI episode was classified as “perioperative” if it occurred within one year of the index surgery, as “hematogenous” if associated with bacteremia, and as "*per continuitatem*" if arising from an adjacent infection (e.g., by fistula formation, infected perigraft hematoma, spondylodiscitis). Surgical treatment was categorized by the most extensive procedure performed within 180 days of VGEI diagnosis, ranging from complete graft excision to debridement or endovascular repair. The Charlson comorbidity index (CCI) assessed the severity of comorbidities [19] while malignancies were reported if active within the past five years.

### Clinical outcome

The primary clinical outcome was all-cause mortality in polymicrobial compared to monomicrobial VGEI, with a maximum follow-up period of 5 years. Patients lost to follow-up were censored at their last recorded timepoint with available survival data.

### Sample collection, processing, and quantitative fitness analysis (QFA) for colony growth dynamics

Tissue and/or graft material from vascular procedures in VGEI patients were collected and processed as described before [20]. A subset of strain pairs from polymicrobial VGEI was selected for this study (convenience sample). QFA was employed to assess reproductive fitness variations among causative pathogens as described [17]. In brief, diluted bacterial cultures were spotted on BHI agar, followed by time-lapse imaging and analysis to define fitness via maximal growth rate and time to maximal growth rate from a Gompertz model fit [17, 20, 21]. Bacterial interactions were studied by comparing strain fitness in co-culture compared to monoculture, thus minimizing the influence of nutrient competition. Strain pairs were drop-plated (3 µl per spot) in a predefined pattern to mimic co-culture (checkerboard of strain 1 and 2) and monoculture. Border culture spots were excluded from analysis to avoid environmental variability (Supplementary Fig. 1).

### Statistical analyses

Clinical data were analyzed to compare patient characteristics and survival between polymicrobial and monomicrobial VGEI. Categorical variables were expressed as numbers and percentages, while quantitative variables were presented as medians and interquartile range (IQR). Fisher’s exact test was used for categorical variables, and the Wilcoxon rank-sum test for continuous variables.

Survival was calculated using Kaplan–Meier analyses, both overall and stratified by infection type (poly- vs. monomicrobial) with the log-rank test comparing groups. Cox proportional hazards models adjusting for age, sex, body mass index (BMI), diabetes mellitus, CCI, and VGEI location were employed. We applied robust standard errors to account for multiple episodes per patient. As a sensitivity analysis, we generated Kaplan–Meier curves and Cox proportional hazards regression for one-year mortality. These analyses were conducted using Stata/SE 18.0 SE (StataCorp, College Station, TX, USA).

Statistical analyses of the QFA data were performed using R 4.4.1 (R Foundation for Statistical Computing, Vienna, Austria, URL https://www.R-project.org/), R Studio, and ggplot2 [22]. Linear mixed effects model was used to evaluate the three-way interaction between strain ID, patient ID and plate layout (mono- versus co-culture) on QFA fitness, incorporating a random intercept for biological replicates [23]. *Post-hoc* estimated marginal means tests were performed for each strain and patient to assess the effect of plate layout based on this model [24].

## Results

### Population characteristics

The analysis included 227 VGEI episodes from 222 patients, of which 81 episodes (35.7%) from 76 patients were classified as polymicrobial. Table 1 summarizes patient characteristics overall and stratified by poly- versus monomicrobial infections. Polymicrobial VGEI were predominantly extracavitary or intracavitary abdominal infections of *per continuitatem* or of perioperative origin, whereas thoracic infections of hematogenous origin were more common in monomicrobial cases. Comorbidities were similar across groups, though peripheral arterial disease and fistula formation were more frequent in patients with polymicrobial infections. Gram-positive bacteria were the most frequently isolated pathogens, followed by Gram-negative and anaerobic bacteria. Fungi were more commonly found in polymicrobial infections (19.8%).

**Table 1:** Baseline characteristics overall and stratified by poly- versus monomicrobial infections (n = 227)

	All VGEI episodes n = 227	Polymicrobial VGEI episodes n = 81	Monomicrobial VGEI episodes n = 146	*p-*value
Female sex, n (%)	49 (21.6)	15 (18.5)	34 (23.3)	0.501
Age median [years] (IQR)	68 (60–76)	68 (60–75)	68.5 (60–76)	0.673
BMI median [kg/m^2^] (IQR)	25.9 (23–28.4)	26.2 (23.3–29)	25.7 (23–28.4)	0.632
Smoking^1^				0.155
Never, n (%)	67 (30.2)	18 (22.5)	49 (34.5)	
Current, n (%)	62 (27.9)	26 (32.5)	36 (25.4)	
Former, n (%)	93 (41.9)	36 (45.0)	57 (40.1)	
Alcohol consumption^2^,				0.662
None/ light n (%)	199 (88.4)	70 (87.5)	129 (88.9)	
Mod/severe, n (%)	26 (11.6)	10 (12.5)	16 (11.0)	
CCI, median (IQR)	2 (1–4)	2 (1–3)	2 (1–4)	0.922
Dyslipidemia, n (%)	144 (63.4)	57 (70.4)	87 (59.6)	0.117
Arterial hypertension, n (%)	176 (77.5)	65 (80.3)	111 (76.0)	0.230
Diabetes mellitus, n (%)	48 (21.2)	18 (22.2)	30 (20.6)	0.865
Diabetes mellitus with end-organ damage^3^, n (%)	14 (6.2)	7 (8.6)	7 (4.8)	0.262
Previous myocardial infarction, n (%)	39 (17.2)	13 (16.1)	26 (17.8)	0.855
Congestive heart failure, n (%)	24 (10.1)	6 (7.4)	18 (12.3)	0.367
Peripheral arterial disease, n (%)	85 (37.4)	42 (51.9)	43 (29.5)	0.001
Cerebrovascular disease, n (%)	51 (22.5)	14 (17.3)	37 (25.3)	0.186
Any malignancy within last 5 years, n (%)	41 (18.1)	19 (23.5)	22 (15.1)	0.149
Location of VGEI, n (%)				< 0.001
Intracavitary abdominal	82 (36.1)	30 (37.0)	52 (35.6)	
Intracavitary thoracic	81 (35.7)	15 (18.5)	66 (45.2)	
Extracavitary	64 (28.2)	36 (44.4)	28 (19.2)	
Route of VGEI, n (%)				< 0.001
Perioperative	62 (27.3)	32 (39.5)	30 (20.1)	
Hematogenous	62 (27.3)	9 (11.1)	53 (36.3)	
*Per continuitatem*	82 (36.1)	38 (46.9)	44 (30.1)	
Mycotic	21 (9.3)	2 (2.5)	19 (13.0)	
Fistula development, n (%)	59 (26.0)	33 (40.7)	26 (17.8)	< 0.001
Graft insertion in an infected site, n (%)	54 (23.8)	18 (22.2)	36 (24.7)	0.746
Pathogens isolated				< 0.001
Anaerobes, n (%)	38 (16.7)	27 (33.3)	11 (7.5)	
Fungi, n (%)	20 (8.8)	16 (19.8)	4 (2.7)	
Gram-negative, n (%)	70 (30.8)	50 (61.7)	20 (13.7)	
Gram-positive, n (%)	140 (61.7)	66 (81.5)	74 (50.7)	
Mycobacteria, n (%)	1 (0.4)	1 (1.2)	0 (0)	
Mycoplasma, n (%)	2 (0.9)	0 (0)	2 (1.4)	
Culture-neg., n (%)	35 (15.4)	0 (0)	0 (0)	
Surgical treatment^4^				< 0.001
Complete excision of graft, n (%)	61 (26.9)	24 (29.6)	37 (25.3)	
Partial excision of graft, n (%)	13 (5.7)	6 (7.4)	7 (4.8)	
Debridement, n (%)	70 (30.8)	38 (46.9)	32 (21.9)	
Endovascular repair, n (%)	3 (1.3)	0 (0)	3 (2.1)	
None, n (%)	80 (35.2)	13 (16.1)	67 (45.9)	

*p*-values calculated with Fisher’s exact test for categorical variables, Wilcoxon rank-sum test for continuous variables

*BMI*, body mass index; *CCI*, Charlson Comorbidity Index; *IQR*, interquartile range; *mod*, moderate; *neg*, negative; *VGEI*, Vascular graft/endograft infection

^1^Missing values for smoking status for 5 episodes, total n = 222

^2^Alcohol consumption was stratified based on the World Health Organization’s definition of light, moderate and severe use. Missing values for alcohol consumption for 2 episodes, total n = 225

^3^End-organ damage defined as: retinopathy, neuropathy, nephropathy

^4^Maximal intervention within 180 days of VGEI diagnosis

### Survival of patients with polymicrobial vs. monomicrobial VGEI

**Fig. 1:**
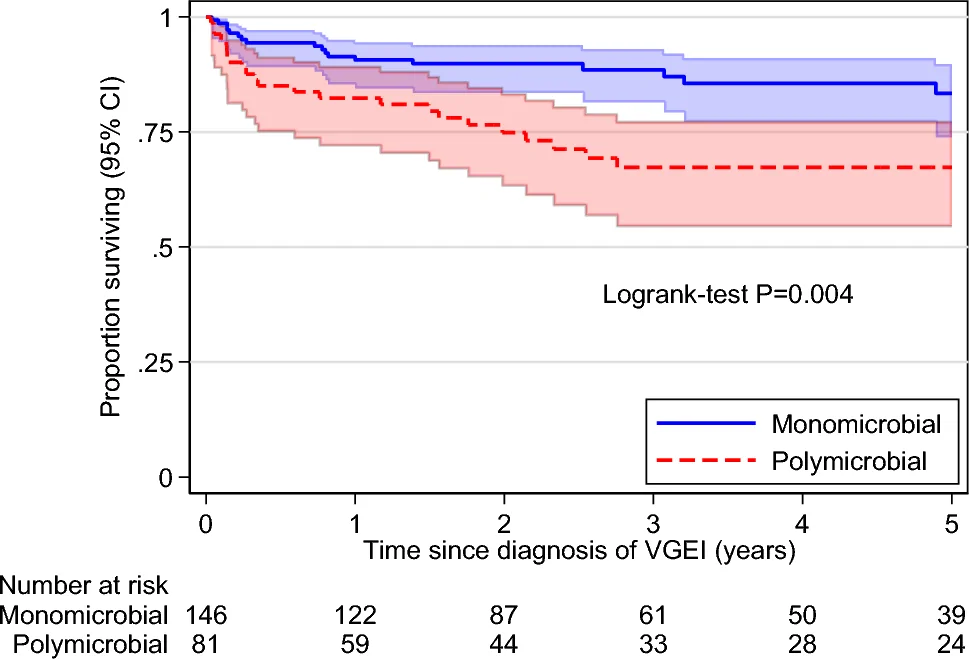
Kaplan–Meier curve for 5-year survival stratified by polymicrobial versus monomicrobial VGEI (n = 227). Unadjusted 5-year survival stratified by poly- versus monomicrobial infection with corresponding 95% Confidence Interval is shown, demonstrating significantly higher mortality in patients with polymicrobial VGEI. Abbreviations: VGEI, vascular graft/endograft infection

The overall survival rate was 87.7% (95% confidence interval [CI]: 82.5–91.4) at one-year and 77.5% (70.2–83.3) at five-years following VGEI diagnosis. Patients with polymicrobial VGEI had lower survival rates (Fig. 1, *p* = 0.004); for one-year mortality, there was also a trend towards reduced survival (Supplementary Fig. 4, *p* = 0.055).

We also examined the association between clinical variables and mortality of VGEI by calculating both unadjusted and adjusted hazard ratios (HRs) for various factors (Table 2). In unadjusted analyses, polymicrobial infections were associated with a higher mortality compared to monomicrobial infections (HR = 2.42, 95% CI: 1.31–4.49, *p* = 0.005). Each 10-year increase in age also increased mortality risk (HR = 1.84, 95% C﻿I: 1.24–2.72, *p* = 0.003). Diabetes mellitus was another significant factor (HR = 2.12, 95% C﻿I: 1.08–4.13, *p* = 0.028). After adjusting for confounders, the association between polymicrobial infection and mortality was stronger (adjusted HR = 2.94, 95% C﻿I: 1.60–5.41, *p* < 0.001). Age remained associated with mortality (adjusted HR = 2.07 per 10 years older, 95% C﻿I: 1.26–3.41, *p* = 0.004). There was also a trend of an association with diabetes mellitus (adjusted HR = 1.95, 95% C﻿I: 0.91–4.29, *p* = 0.088). Other factors, including sex, BMI and CCI, had no significant effect on mortality in the adjusted models. HR for one-year mortality were comparable across groups and are presented in Supplementary Table 1.

**Table 2:** Results of unadjusted and adjusted Cox proportional hazards models for death

Variable	Unadjusted			Adjusted^1^		
	HR	95% CI	*p*-value	HR	95% CI	*p*-value
Monomicrobial	ref			ref		
Polymicrobial	2.42	1.31–4.49	0.005	2.94	1.60–5.41	< 0.001
Male	ref			ref		
Female	0.65	0.27–1.56	0.333	0.64	0.24–1.67	0.360
Age, per 10 years	1.84	1.24–2.72	0.003	2.07	1.26–3.41	0.004
BMI						
< 25 kg/m^2^	ref			ref		
25–30 kg/m^2^	0.77	0.38–1.57	0.480	0.64	0.29–1.41	0.265
> 30 kg/m^2^	1.15	0.53–2.50	0.714	1.14	0.46–2.82	0.781
CCI > 4	1.87	0.94–3.72	0.075	1.64	0.82–3.30	0.164
Location						
Intracavitary abdominal	ref			ref		
Intracavitary thoracic	0.87	0.41–1.83	0.706	1.49	0.68–3.27	0.323
Extracavitary	1.26	0.60–2.62	0.546	0.90	0.39–2.10	0.804
Smoking						
Never	ref					
Past	1.18	0.59–2.38	0.641			
Current	0.62	0.26–1.48	0.282			
Active malignancy^2^	1.67	0.85–3.27	0.138			
Coronary heart disease or heart failure	1.73	0.90–3.32	0.102			
Diabetes mellitus	2.12	1.08–4.13	0.028	1.95	0.91–4.29	0.088
Peripheral arterial disease	1.04	0.55–1.96	0.912			
Fistula development	0.76	0.36–1.59	0.469			
Graft insertion in an infected site	1.51	0.77–2.97	0.232			
Maximal intervention within 180 days						
None	ref					
Endovascular repair	4.23	0.45–39.4	0.206			
Debridement	1.64	0.74–3.63	0.220			
Partial graft removal	1.35	0.32–5.77	0.682			
Complete graft removal	1.90	0.84–4.31	0.126			

*BMI*, body mass index; *CCI*, Charlson Comorbidity Index; *CI*, Confidence interval; *HR*, Hazards ratio

Total n = 227

^1^Adjusted for sex, age, BMI, CCI, graft location

^2^Defined as: Malignancy diagnosed or treated within the last 5 years (solid or hematologic)

### Quantification of fitness of different pathogens when grown in co-culture

A total of 137 clinical samples were obtained from patients with VGEI. Five cases of VGEI with two concurrent causative pathogens were selected for QFA (Table 3). The clinical course of these five patients is detailed in the supplementary material (Case descriptions and Supplementary Figs. 2 and 3a).

**Table 3:** Summary of clinical isolates and corresponding clinical information of 5 patients with polymicrobial VGEI

Patient Nr	Bacterial species	Sex	Age	IVDU	Sample material	Removal of prosthesis	ICU stay	Bacteraemia (days)	Antibiotic treatment	Death
P1	*C. amycolatum* *S. aureus*	M	60	Yes	Deep wound tissue	Yes	No	None	TZP VA DAP CIP MTZ CN	No
P2	*C. glabrata* *E. coli*	M	72	No	Graft prosthesis	Yes	Yes	None	CAS TZP MEM DAP VA	Yes
P3	*K. aerogenes* *C. glabrata*	M	53	Yes	Deep wound tissue	No	No	Yes (10)	AMC VA CIP OX DAP CN ISA SXT RA	No
P4	*E. faecalis* *C. amycolatum*	M	51	No	Deep wound tissue	No	Yes	None	AMC	Yes
P5	*S. epidermidis* *E. cloacae*	M	74	No	Deep wound tissue	No	Yes	None	MEM VA	No

*M*, Male; *IVDU*, Intravenous drug use; *ICU*, intensive care unit; *C. amycolatum, Corynebacterium amycolatum; C. glabrata, Candida glabrata; E. cloacae, Enterobacter cloacae; E. faecalis, Enterococcus faecalis; E. coli, Escherichia coli; K. aerogenes, Klebsiella aerogenes; S. aureus, Staphylococcus aureus; S. epidermidis, Staphylococcus epidermidis*; *AMC*, Amoxicillin-clavulanic acid; *CAS*, Caspofungin; *CIP*, Ciprofloxacin; *SXT*, Trimethoprim/Sulfamethoxazol; *DAP*, Daptomycin; *OX*, Flucloxacillin; *CN*, Gentamicin; *ISA*, Isavuconazol; *MTZ*, Metronidazol; *MEM*, Meropenem; *TZP*, Piperacillin/Tazobactam; *RA*, Rifampicin; *VA*, Vancomycin

**Fig. 2:**
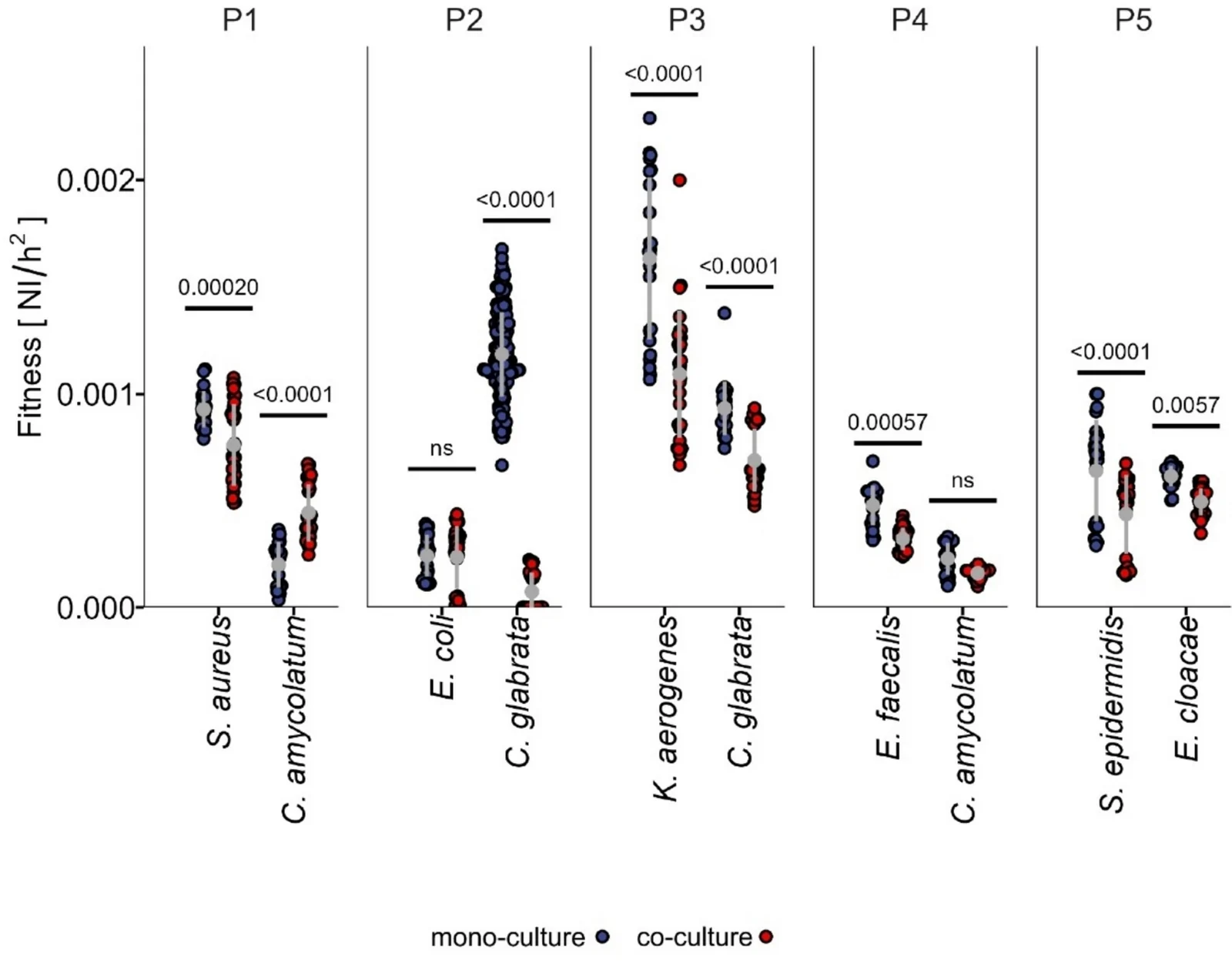
Fitness of five strain pairs from five patients with polymicrobial VGEI in mono- vs. co-culture. Plate growth was tracked using time-lapse imaging over a 48-h period, and the absolute fitness was subsequently calculated. The absolute fitness of all pathogens was assessed in mono- (blue) and in co-culture with the corresponding second isolate from each strain pair (red). Each data point represents a single technical replicate, and the mean ± standard deviation is indicated for each pathogen and layout (mono- versus co-culture). *p*-values for each pathogen are for interaction effect of mono- vs. co-culture assessed with a linear mixed-effect model. We used separate culture plates for *C. glabrata* monoculture (P2), as the strong growth inhibiting effect of *E. coli* did not allow to assess this condition on the same plate. Abbreviations: NI, normalized intensity

Using QFA, we quantified the reproductive fitness of five polymicrobial isolate pairs, revealing diverse interaction patterns among the strain pairs (Fig. 2). In patient 1, the growth of *Corynebacterium amycolatum* was enhanced by *S. aureus*, while *S. aureus* growth was inhibited by *C. amycolatum*, indicating a substantial interaction (*p*-value < 0.001; Supplementary Fig. 2). In patient 2, *Candida glabrata* was completely inhibited by *Escherichia coli*, while *E. coli* fitness remained unaffected by *C. glabrata.* Notably, *C. glabrata* did not grow on control spots, suggesting a potent inhibitory effect of *E. coli.* This was confirmed by an inhibition assay, where plating the two strains at increasing distances showed a decrease in inhibition with greater distance (Supplementary Fig. 3b/c) [22]. Co-culture experiments with *Klebsiella aerogenes* and *Candida glabrata, Enterococcus faecalis* and* Corynebacterium amycolatum,* and* Staphylococcus epidermidis* and* Enterobacter cloacae* (patient 3, 4 and 5) all demonstrated significantly impaired growth of one or both isolates.

## Discussion

In this analysis of 227 VGEI episodes, we aimed to better characterize different aspects of polymicrobial infections. After adjustment for age and comorbidities, patients with polymicrobial infections exhibited an almost 2.5-fold increase in one-year mortality and a threefold increase in five-year mortality risk. From a microbiological perspective, QFA of five pathogen pairs from polymicrobial infections demonstrated to be a cost-effective method for revealing diverse interaction patterns. We primarily identified negative interactions (one- or two-sided inhibition), although some neutral or positive (one-sided) changes in fitness were also observed when comparing mono- to polymicrobial growth.

Comparing our clinical results with age-matched cohorts with similar comorbidities remains challenging due to the rarity of VGEI. The one-year mortality rate in cohorts of patients with VGEI varies by infection site and management strategy. A 2025 observational cohort found a one-year all-cause mortality of 32.5% with higher mortality in VGEI not undergoing surgery [25]. Another retrospective study reported one-year mortality rates of 12.5% for thoracic-aortic graft infections, 55.6% for abdominal-aortic graft infections, and 30% for peripheral-arterial graft infections, highlighting the impact of infection location [26]. A recent French–English cohort [27] including only patients deemed unfit for surgery reported one-year mortality rates of 29.6% and five-year mortality rates of 56.9%.

Recent studies on polymicrobial *S. aureus* bacteraemia report higher mortality rates in polymicrobial infections [12, 28]. Observational studies on infective endocarditis in people who inject drugs [13] and a study in U.S. veterans [29], also suggest that polymicrobial infections are associated with increased complications and worse clinical outcomes. Polymicrobial infections and increasing age were consistently associated with a higher risk of mortality in both the unadjusted and adjusted models. Among frequent comorbidities, diabetes mellitus was associated with higher mortality, underscoring the importance of optimal management to improve patient outcomes. While the effect of factors such as sex, BMI, and smoking status on mortality remained uncertain, it is important to note that these variables may still have a role in influencing other clinical aspects of VGEI. Further studies with larger sample sizes may help clarify their potential indirect effects on mortality.

Investigating pathogen interactions in polymicrobial infections could offer valuable insights into the pathophysiological processes contributing to poorer outcomes. Our study showed a reduction of fitness of *S. aureus* in the presence of *C. amycolatum*, consistent with existing literature on the impact of *Corynebacterium* spp. on *S. aureus* colonization, viability, and virulence [14, 15, 30]. Microbe-microbe interactions, especially in the context of microbiota and infections, have been studied using various methods. Ramsey [15] used RNA sequencing to assess gene expression in *S. aureus* during mono- or co-culture with *C. striatum*, showing upregulation of genes related to nasal colonization and downregulation of invasive-associated genes. Additionally, *S. aureus’s* haemolytic capacity was reduced after exposure to *C. striatum* cell-free conditioned medium. Another study demonstrated inhibitory activity against *S. aureus* by *C. amycolatum* through zone of clearance measurements on agar plates in both contact-dependent and -independent assays [14].

Further studies have explored microbe-microbe interactions by examining the effect of *S. aureus* on the mucosal barrier, with and without *Corynebacterium* supernatant, using confocal microscopy to assess barrier integrity. In a mouse subcutaneous abscess model, colony-forming units (CFU) from mono- and coinfection abscesses of *S. aureus* and *C. striatum* were counted, showing a significant reduction in *S. aureus* CFUs in the presence of *C. striatum* [14]. Interestingly, our patient with *S. aureus* and *C. amycolatum* infection had a chronic, exuding wound from an infected vascular graft, suggesting that *S. aureus* may shift toward a more commensal behaviour in the presence of C*. amycolatum* [15].

We quantified the inhibitory effect of *E. coli* on *C. glabrata*, which aligns with existing literature demonstrating *E. coli'*s inhibitory effects on various *Candida* species. Bandara showed that *E. coli* secretory products significantly inhibited biofilm formation in six *Candida* spp. strains [31]. Similarly, Cabral reported a fungicidal effect from both *E. coli* co-culture and *E. coli*-conditioned media on *C. albicans,* while *E. coli*’s growth remained unaffected, suggesting the secretion of a fungicidal element [32]. Although no studies have specifically examined the interaction between *K. aerogenes* and *C. glabrata*, research involving caterpillar larvae suggests a synergistic pathogen-pathogen interaction in the biofilm of *K. aerogenes* and *C. albicans* [33]. Such synergy might be mediated by enhanced biofilm formation, altered quorum sensing, and metabolic crosstalk.

Although we primarily observed antagonistic interactions between isolated strains, patients with polymicrobial infections experienced higher mortality rates, even after adjustment for age, graft location, and comorbidities. Several factors may contribute to this increased mortality risk, including the need for more toxic combination therapies, and complex interactions at both the microbe-microbe and microbe-host levels. Pathogen competition could result in persistent low-grade infections that are difficult to eradicate and may lead to altered inflammatory responses.

Our study has several strengths, including the prospective enrolment of patients with incident VGEI and the use of patient-derived bacterial strains. To the best of our knowledge, this is the largest analysis of patient characteristics and outcomes in polymicrobial VGEI. We assessed the fitness of a wide range of pathogens from polymicrobial infections using solid agar to replicate the graft infection environment. By comparing mono- and co-cultures, we ruled out nutrient limitations as a factor affecting growth. Additionally, QFA enabled the measurement of full growth curves, including culture doubling time. Notably, QFA is cost-effective compared to other methods as it does not require fluorescence staining or microscopy and eliminates the need for time-consuming CFU-counting [15, 34].

Our study has limitations. In the fitness analysis, we focused on a random subset of patient samples from a specific timeframe, without accounting for patient-specific factors. While the microbiological rationale for including diverse pathogens is clear, the clinical implications of observed fitness changes remain speculative. Our sampling strategy captured only a single timepoint in the infection process, limiting QFA's ability to discern the longitudinal impact of pathogen interactions. Despite these limitations, our approach provides qualitative insights into polymicrobial interactions.

In conclusion, our study establishes an analytical framework and an affordable method for quantifying polymicrobial interactions, while also paving the way for future research into the complex relationship between polymicrobial infections and clinical outcomes. Expanding this study to a larger scale with more patient samples and time points could provide valuable quantitative data. Future research using QFA has the potential to offer a deeper insight into pathogen interactions and their impact on clinical outcomes. Understanding the in vitro behaviour of pathogens may ultimately inform personalized patient management strategies.

## Supplementary Information

Below is the link to the electronic supplementary material.

Supplementary file1 (DOCX 5655 KB)

## Data Availability

Data is provided within the manuscript or supplementary information files.
