# Supplementary material for: Clinical and microbial insights into polymicrobial vascular graft infections: a prospective observational study
Source: Infection. 2026 Mar 26;54(3):1427–35. doi: 10.1007/s15010-026-02775-1 (PMC13661717; doi:10.1007/s15010-026-02775-1)
Supplement: Supplementary file 1 [file 15010_2026_2775_MOESM1_ESM.docx]

# SUPPLEMENTARY MATERIAL

# Detailed clinical histories of the five patients with polymicrobial VGEI and QFA analysis

**Patient 1**: A 60-year-old male intravenous drug user with chronic hepatitis C was transferred to the University Hospital Zurich (USZ) due to a VGEI involving his iliac-femoral obturator bypass. He presented with a persistent, chronically exuding wound in the right groin. The collagen-coated vascular graft, originally implanted in 2006 to address an infected false aneurysm, had been occluded since 2011 despite multiple revisions. On arrival at USZ, a CT scan revealed a prosthetic-cutaneous fistula and an abscess surrounding the bypass, necessitating explantation of the graft. Cultures from the pus around the surgical site and the explanted bypass identified *Staphylococcus aureus* and *Corynebacterium amycolatum* (**strain pair from patient 1**). Due to good collateralization, it was not necessary to implant a new graft to ensure perfusion to the leg. The patient underwent a 20-day course of amoxicillin-clavulanic acid and was discharged without any signs of relapse or reinfection. Clinical and laboratory follow-up to date has confirmed the absence of recurrence. **Supplementary Figure 2** provides a graphical depiction of the patient’s clinical course.

**Patient 2:** In 2021, a 72-year-old male was hospitalized for a suspected relapse of VGEI, following a two-months history of recurrent fever, chills, and abdominal pain. His medical history included graft replacement due to chronic VGEI caused by *Pseudomonas aeruginosa*, with a residual ingrown vascular graft. Post replacement, targeted therapy for Pseudomonas was maintained for one year. Upon admission in 2021, blood cultures identified *Pseudomonas aeruginosa*, *Escherichia coli*, and *Prevotella* spp., prompting treatment with piperacillin/tazobactam. The infected abdominal graft was replaced with a bovine pericardial prosthesis. Cultures from the explanted graft yielded *C. glabrata* and *E. coli* (**strain pair from patient 2**). The patient’s hospital course was marked by multiple complications, including mesenteric ischemia necessitating hemicolectomy. Despite aggressive treatment, the patient succumbed after a prolonged and challenging 96-day course in intensive care. **Supplementary Figure 3a** illustrates the patient’s clinical progression.

**Patient 3:** A 53-year-old male with HIV/hepatitis C-coinfection and a history of intravenous drug use was hospitalized with swelling and bleeding in his right groin. He was diagnosed with a femoral artery aneurysm and underwent xenopericardial patchplasty. Tissue and blood cultures grew Methicillin-resistant *Staphylococcus aureus* (MRSA), prompting treatment with vancomycin, which was later switched to daptomycin and flucloxacillin due to persistent bacteraemia. Despite antimicrobial treatment, the inguinal wound dehisced, exposing the vascular graft. Subsequent debridement identified *Klebsiella aerogenes* and *Candida glabrata* (**strain pair from patient 3**). The wound was managed with closure using a sartorius muscle flap. The patient was discharged after six days and prescribed a 12-week regimen of trimethoprim/sulfamethoxazole, rifampicin, and isavuconazole.

**Patient 4** A 51-year-old male on hemodialysis was transferred to the hospital with elevated inflammatory markers (CRP 301 mg/L, Lc 21 G/L). Examination revealed an exposed polytetrafluoroethylene (PTFE) brachio-axillary shunt, strongly suggestive of another infectious complication following two years of treatment with clindamycin and ciprofloxacin for previous infections caused by *S. aureus* and *Citrobacter freundii*. Emergency surgery was performed to remove the exposed shunt material, and the antimicrobial treatment was switched to vancomycin and meropenem. Cultures from the graft identified *Enterococcus faecalis* and *Corynebacterium amycolatum* (**strain pair from patient 4**). Unfortunately, the patient developed uncontrollable gastric bleeding, resulting in death from haemorrhagic shock on the fourth day of hospitalization.

**Patient 5:** A 73-year-old male underwent aortic graft implantation for a newly diagnosed thoracoabdominal aortic aneurysm. Postoperatively, he experienced haemorrhagic shock due to bleeding from the common iliac vein, necessitating an emergency relaparotomy. On the 10^th^ postoperative day, ischemic perforation of the sigmoid colon required hemicolectomy. During the procedure, a 2x5 cm peritoneal rupture was discovered, exposing the retroperitoneal vascular prosthesis indicating secondary VGEI. Cultures from abdominal and retroperitoneal swabs identified multi-resistant *E. cloacae* AmpC and methicillin-resistant *S. epidermidis* (**strain pair from patient 5**), prompting a change in antimicrobial therapy to daptomycin and ceftazidime/avibactam. Following discharge, the patient continued a six-month course of antimicrobial treatment, and no further infectious episodes have been reported.

# Supplementary Figure 1: Individual growth curves derived from time-lapse images

**Panel A**

**Panel B**

**Panel A.** General QFA setup. Strain-couplets were drop-plated in a predefined pattern, with strains growing in a checkerboard-pattern at the top to create a co-culture of strain 1 and 2, while at the bottom strains grew in monoculture. The analysis excluded borders of each condition, including solely the spots within the yellow frames in further analysis.

**Panel B**. Calculated growth curves per spot.

# Supplementary Figure 2: Clinical course of a VGEI due to *S. aureus* and *C. amycolatum* (patient 1)

**2: Clinical course**

**Figure legend:** An overview of the clinical course, antimicrobial treatments, surgical interventions, and pathogen sampling relevant to the vascular graft/ endograft infection.

Abbreviations: NPWT, negative pressure wound therapy.

# Supplementary Figure 3: Clinical course of a VGEI due to *E. coli* and *C. glabrata* (Patient 2)

**3a: Clinical course**

**3b: QFA-setup for *E. coli* and *C. glabrata*, showing almost no growth of *C. glabrata***

**3c: Inhibition assay**

**Figure legend**

**3a:** Summary of the clinical course, antimicrobial and surgical treatments, and pathogen sampling relevant to the vascular graft/ endograft infection.

**3b:** QFA-setup of *E. coli* (growth visible on bottom right side in monoculture and in coculture on the top half of the agar plate) and *C. glabrata* (minimal growth on bottom left side and no growth visible in grid). The calculation of fitness was not possible due to the lack of growth of *C. glabrata* in the control spots. We postulated, that *E.* coli managed to inhibit *C. glabrata* on the whole agar plate. As a result, we attempted to assess the inhibitory effect of *E. coli* on *C. glabrata* using an inhibition assay.

**3c:** Results of inhibition assay between *E. coli* and *C. glabrata*. *C. glabrata* exhibited better growth as distance from the *E. coli spots* increased. Since no competition was observed between the *E. coli* spots, this effect is likely due to the inhibitory action of *E. coli* on *C. glabrata,* rather than nutrient depletion in the agar. As shown in the image on the right, the inhibitory effect was more pronounced and covered a wider area when *E. coli* was plated 24h earlier. This suggests that inhibitory factors from *E. coli* had more time to diffuse into the agar.

# Supplementary Figure 4: Kaplan Meier curve for one-year survival stratified by polymicrobial versus monomicrobial VGEI (n=227)

**Figure legend:** Unadjusted one-year survival stratified by poly- versus monomicrobial infection with corresponding 95% Confidence Interval.

Abbreviations: VGEI, vascular graft/endograft infection.
**Supplementary Table 1: Results of unadjusted and adjusted Cox proportional hazards models for one-year mortality**

| **Variable** | **Unadjusted** | | | **Adjusted ^1^** | | |
| --- | --- | --- | --- | --- | --- | --- |
|  | **HR** | **95% CI** | ***P*-value** | **HR** | **95% CI** | ***P*-value** |
| **Monomicrobial** | ref |  |  | ref |  |  |
| **Polymicrobial** | 2.06 | 0.97-4.36 | 0.06 | 2.48 | 1.18-5.21 | 0.017 |
| **Male** | ref |  |  | ref |  |  |
| **Female** | 0.64 | 0.22-1.86 | 0.409 | 0.67 | 0.21-2.15 | 0.506 |
| **Age, per 10 years** | 2.03 | 1.21-3.42 | 0.008 | 2.26 | 1.12-4.24 | 0.024 |
| **BMI** |  |  |  |  |  |  |
| <25 kg/m^2^ | ref |  |  | ref |  |  |
| 25-30 kg/m^2^ | 0.93 | 0.41-2.12 | 0.864 | 0.69 | 0.27-1.78 | 0.445 |
| >30 kg/m^2^ | 0.79 | 0.26-2.41 | 0.680 | 0.73 | 0.20-2.61 | 0.624 |
| **CCI >4** | 1.95 | 0.85-4.46 | 0.113 | 1.58 | 0.68-3.65 | 0.289 |
| **Location** |  |  |  |  |  |  |
| Intracavitary abdominal | ref |  |  | ref |  |  |
| Intracavitary thoracic | 1.02 | 0.40-2.57 | 0.975 | 1.73 | 0.59-5.05 | 0.317 |
| Extracavitary | 1.33 | 0.53-3.36 | 0.541 | 0.86 | 0.32-2.29 | 0.768 |
| **Smoking** |  |  |  |  |  |  |
| Never | ref |  |  |  |  |  |
| Past | 1.33 | 0.56-3.16 | 0.519 |  |  |  |
| Current | 0.67 | 0.22-2.01 | 0.470 |  |  |  |
| **Active malignancy ^2^** | 1.0 | 0.38-2.65 | 0.993 |  |  |  |
| **Coronary heart disease or heart failure** | 1.47 | 0.67-3.21 | 0.337 |  |  |  |
| **Diabetes mellitus** | 3.32 | 1.56-7.18 | 0.002 | 3.08 | 1.25-7.60 | 0.014 |
| **Peripheral arterial disease** | 1.41 | 0.66-3.01 | 0.376 |  |  |  |
| **Fistula development** | 0.65 | 0.24-1.72 | 0.383 |  |  |  |
| **Graft insertion in an infected site** | 1.68 | 0.75-3.74 | 0.208 |  |  |  |
| **Maximal operation within 180 days** |  |  |  |  |  |  |
| None | ref |  |  |  |  |  |
| Endovascular repair | 5.87 | 0.76-45.3 | 0.090 |  |  |  |
| Debridement | 1.54 | 0.58-4.11 | 0.383 |  |  |  |
| Partial graft removal | 0.92 | 0.11-7.42 | 0.935 |  |  |  |
| Complete graft removal | 1.87 | 0.76-4.95 | 0.212 |  |  |  |

**Abbreviations**: BMI, body mass index; CCI, Charlson Comorbidity Index; CI, Confidence interval; HR, Hazards ratio.

**Notes**:

Total n=227.

1 Adjusted for sex, age, BMI, CCI, graft location, diabetes mellitus

2 Defined as: Malignancy diagnosed or treated within the last 5 years (solid or hematologic).
